# Soluble Uric Acid Activates the NLRP3 Inflammasome

**DOI:** 10.1038/srep39884

**Published:** 2017-01-13

**Authors:** Tarcio Teodoro Braga, Maria Fernanda Forni, Matheus Correa-Costa, Rodrigo Nalio Ramos, Jose Alexandre Barbuto, Paola Branco, Angela Castoldi, Meire Ioshie Hiyane, Mariana Rodrigues Davanso, Eicke Latz, Bernardo S. Franklin, Alicia J. Kowaltowski, Niels Olsen Saraiva Camara

**Affiliations:** 1 Laboratory of Transplantation Immunobiology, Department of Immunology, Institute of Biomedical Sciences IV, University of São Paulo (USP), São Paulo, Brazil; 2 Institute of Innate Immunity, University Hospital, University of Bonn, Bonn, Germany; 3 Departamento de Bioquímica, Instituto de Química, USP, São Paulo, Brazil; 4 Laboratory of Tumor Immunology Department of Immunology, Institute of Biomedical Sciences IV, University of São Paulo (USP), São Paulo, Brazil; 5 Department of Cellular Biology - Institute of Biomedical Sciences, University of São Paulo (USP), São Paulo, Brazil; 6 Division of Infectious Diseases & Immunology, University of Massachusetts Medical School, Worcester, Massachusetts, USA; 7 German Center for Neurodegenerative Diseases, Bonn, Germany; 8 Center of Molecular Inflammation Research, Department of Cancer Research and Molecular Medicine, Norwegian University of Science and Technology, Trondheim, Norway; 9 Laboratory of Clinical and Experimental Immunology, Nephrology Division, Federal University of São Paulo (UNIFESP), São Paulo, Brazil; 10 Renal Pathophysiology Laboratory (LIM16), Faculty of Medicine, University of São Paulo, Brazil

## Abstract

Uric acid is a damage-associated molecular pattern (DAMP), released from ischemic tissues and dying cells which, when crystalized, is able to activate the NLRP3 inflammasome. Soluble uric acid (sUA) is found in high concentrations in the serum of great apes, and even higher in some diseases, before the appearance of crystals. In the present study, we sought to investigate whether uric acid, in the soluble form, could also activate the NLRP3 inflammasome and induce the production of IL-1β. We monitored ROS, mitochondrial area and respiratory parameters from macrophages following sUA stimulus. We observed that sUA is released in a hypoxic environment and is able to induce IL-1β release. This process is followed by production of mitochondrial ROS, ASC speck formation and caspase-1 activation. *Nlrp3*^*−/−*^ macrophages presented a protected redox state, increased maximum and reserve oxygen consumption ratio (OCR) and higher VDAC protein levels when compared to WT and *Myd88*^*−/−*^ cells. Using a disease model characterized by increased sUA levels, we observed a correlation between sUA, inflammasome activation and fibrosis. These findings suggest sUA activates the NLRP3 inflammasome. We propose that future therapeutic strategies for renal fibrosis should include strategies that block sUA or inhibit its recognition by phagocytes.

Uric acid, the product of purine catabolism, is a damage-associated molecular pattern (DAMP) released from ischemic tissues and dying cells^1^,^2^. Once crystalized, uric acid activates the immune system^3^,^4^, since it acts as a pro-oxidant molecule that reduces nitric oxide availability^1^, increases the production of reactive oxygen species (ROS), stimulates chemotaxis and also activates NF-κB and MAPK pathways^1^. Uric acid crystals also induce the release of proinflammatory cytokines such as IL-1β^4^,^5^, a signaling molecule secreted when the formation of a high molecular weight complex named “inflammasome” is activated^6^.

Crystals trigger inflammasome activation^4^,^7^,^8^,^9^ mainly through frustrated phagocytosis, a process characterized either by aberrant actin polymerization^10^ or by lysosomal damage^11^, in which lysosome contents leak into the cytosol. Lysosomal proteases, once in the cytosol, in addition to acting as DAMPs^12^, are able to digest vital proteins and affect other organelles, such as the mitochondria. In addition, uric acid crystals can directly engage cellular membranes without the involvement of any known cellular receptor. Recent evidences demonstrate that serum uric acid levels are also associated with inflammatory effects, such as in gout-related disease and preeclampsia^13^,^14^. However, it is still unknown if soluble uric acid (sUA) activates the inflammasome complex and lead to IL-1β production. Primates lost the uricase gene and the direct consequence is that they have higher serum uric acid levels than other animals^15^. The mutation in the uricase gene that occurred during food scarcity and global cooling resulted in a survival advantage at that time^16^. Today, however, it is associated with hypertension, kidney disease, obesity and diabetes^17^. Accumulating evidence has revealed a positive relationship between serum uric acid levels and cardiovascular mortality in patients with chronic kidney disease^18^,^19^. Despite an unknown mechanism, local accumulation of uric acid may activate the inflammasome complex, leading to inflammation and fibrosis^20^.

The most studied inflammasome sensor is the NACHT, LRR, and PYD domain-containing protein 3 (NLRP3), a protein that interacts with the adapter molecule, ASC, which in turn interacts with pro-caspase-1^21^,^22^. A specific gain-of-function mutation in the *Nlrp3* gene is a marker of auto inflammatory disorders^23^,^24^. In affected patients, mutations in *Nlrp3* lead to a spontaneous release of IL-1β^25^. NLRP3 activation has been shown to play important roles in many auto inflammatory^26^,^27^, metabolic^28^,^29^,^30^ and neurodegenerative diseases^31^,^32^. ROS production in macrophages triggers the NLRP3 inflammasome^9^,^33^, but the cellular compartment from which ROS originates remains controversial^34^. The main source of a diverse variety of ROS in most cells is mitochondria^35^,^36^, in which a large number of oxidant-generating reactions exist^37^. ROS act as signaling molecules and regulate metabolism, proliferation^38^ and inflammation, as indicated by the fact that either deficiency in NADPH oxidase (an enzymatic ROS source) or the presence of antioxidants inhibit NLRP3 inflammasome activation^9^. In addition to ROS production, mitochondria orchestrate NLRP3 inflammasome activation through mechanisms involving changes in mitochondrial DNA^39^, membrane lipids^40^ and ion flux^41^, among others^42^.

In the present study, we investigated whether uric acid could induce the production of IL-1β in its soluble form. We hypothesized that sUA is able to activate the NLRP3 inflammasome. We monitored ROS production, modifications in mitochondrial area and respiratory parameters from macrophages following sUA stimulus and observed that sUA is released in a hypoxic environment and, when the first signal of inflammasome activation is present, is able to trigger NLRP3 through the production of mitochondrial ROS. This process is followed by ASC speck formation, caspase-1 activation and IL-1β production. We took advantage of a fibrotic disease model in which increased levels of sUA occur, and observed a correlation between tissue damage and the degree of sUA formation. Indeed, we demonstrate that macrophages derived from *Nlrp3* deficient mice, when compared to WT and *Myd88* deficient animals, present protected redox state, increased maximum and reserve oxygen consumption rates (OCR), and higher VDAC protein levels.

## Results

### Soluble Uric Acid Activates the NLRP3 Inflammasome

**Figure 1:**
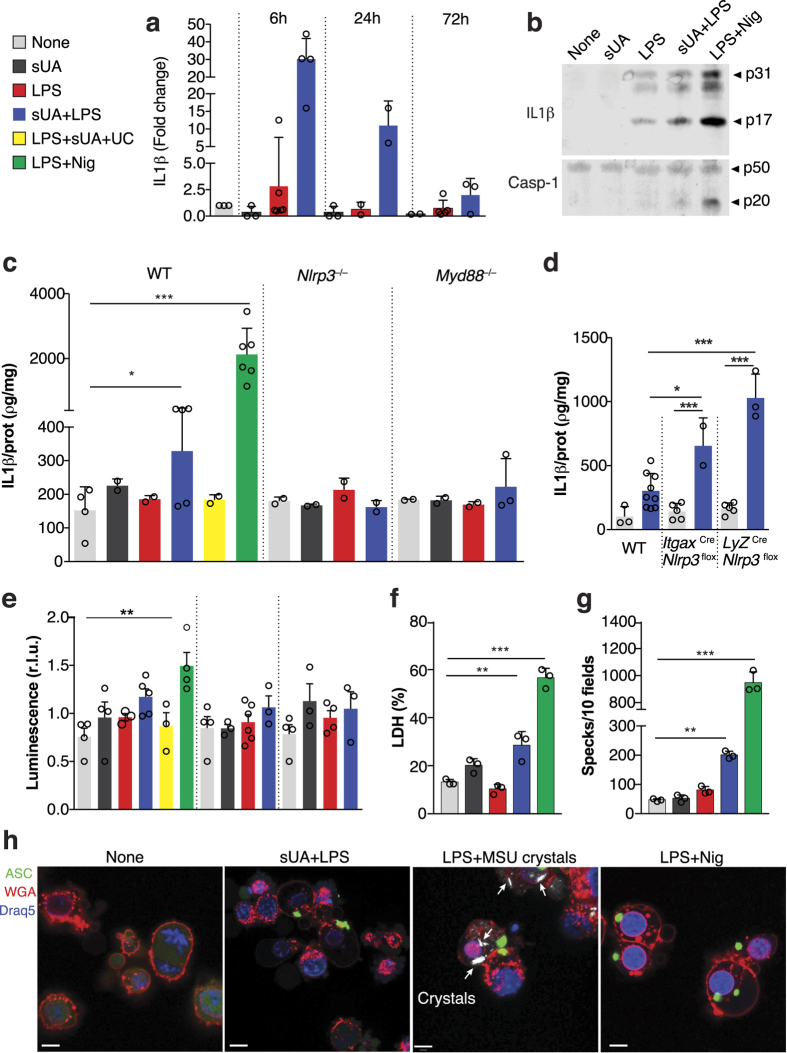
Soluble uric acid stimulates IL-1β production in a NLRP3- and MyD88-dependent way. Bone marrow-derived macrophages were stimulated with sUA (180 μΜ) and LPS (10 ηg/mL). Non-stimulated macrophages were used as controls and uricase (1 U/mL) and nigericin (10 μΜ) were used in some experiments. (**a**) *il-1β* mRNA was analyzed at different time points. (**b**) Western blotting of IL-1β and caspase-1 of cell supernatants after 24 hours stimulus. (**c**) Elisa of IL-1β of supernatants of WT, NLRP3^−/−^ and MyD88^−/−^ macrophages after different stimuli for 24 h. (**d**) Elisa of IL-1β was analyzed in supernatants of WT, Itgax^Cre^NLRP3^flox^ and LyZ^Cre^NLRP3^flox^ macrophages after sUA+LPS stimulation for 24 h. (**e**) WT, NLRP3^−/−^ and MyD88^−/−^ macrophages were transfected with a luciferase-based inflammasome and protease activity reporter for proteolytic activity of caspase-1 (iGLuc). Luciferase activity was assessed 96 h after the indicated stimulus in the cellular supernatants. (**f**) Release of lactate dehydrogenase (LDH) into supernatants was measured at 24 hours and shown as percentage of maximum at each stimulus. (**g**) Immortalized ASC-mCerulean macrophages were cultivated for 24 hours with indicated stimulus and ASC specks are represented as number of specks per ten microscopy fields. (**h**) Representative confocal photomicrographs of ASC-mCerulean macrophages showing ASC specks (green) produced after the activation of inflammasomes. The plasmatic membrane is indicated in red, the nucleus in blue and crystals are shown by white arrows. In (**a**), qPCR data were normalized to HPRT expression, and the mean of the control condition was considered 1. In (**b**), the blots are cropped in order to improve the clarity and conciseness of the membrane presentation. In (**e**), r.l.u. indicates relative light units. In (**h**), the white bars represent 50 μm. Data are representative of two independent experiments. n = 2 to 9. *p < 0.05; **p < 0.01; and ***p < 0.001. Nig = Nigericin; UC = uricase.

We incubated bone marrow-derived macrophages in the presence of soluble uric acid alone (sUA) and/or LPS (sUA+LPS) for 6, 24 or 72 hours. We observed that SUA+LPS induced IL-1β mRNA expression when compared to non-stimulated cells and to cells stimulated with either sUA, or LPS alone. IL-1β expression was higher at 6 h and decreased with time (Fig. 1a). Cleaved IL-1β levels were enhanced in the supernatant after SUA+LPS stimulation for 24 h (Fig. 1b). We also observed that inflammasome activation by sUA+LPS is NLRP3- and MyD88-dependent, since macrophages from *Nlrp3*^−/−^ and *Myd88*^−/−^ mice failed to produce IL-1β levels comparable to Wild-type mice (WT) (Fig. 1c). Moreover, as shown in Supplementary Fig. 1 A, sUA stimulates IL-1β production in a dose-dependent way and the cytokine levels are lower than that observed after monosodium urate (MSU) crystals stimulus. Macrophages with a gain of function of *Nlrp3* under a lysozyme (*Lyz*^*Cre*^*Nlrp3*^*flox*^) or integrin alpha X (*Itgax*^*Cre*^*Nlrp3*^*flox*^) promoter presented increased levels of IL-1β after sUA+LPS stimulus when compared to WT macrophages (Fig. 1d). Inflammasome activation with sUA+LPS was further confirmed through the measurement of the proteolytic activity of caspase-1. We observed reduced caspase-1 activity in *Nlrp3*^−/−^ and *Myd88*^−/−^ macrophages when compared to WT (Fig. 1e). To confirm that IL-1β production was due to the presence of uric acid promoted by LPS, we added uricase (UC) into WT macrophage cultures right before stimulating them with sUA+LPS. The decreased levels of IL-1β protein (Fig. 1c) and decreased caspase-1 activity (Fig. 1e) observed confirm that uric acid is responsible for these effects. Like many other inflammasome activators, sUA+LPS stimulation results in pyroptosis, as measured by the release of lactate dehydrogenase (LDH) (Fig. 1f). sUA also induced the polymerization of the adaptor molecule ASC and the assembly of ASC specks when added in the presence of LPS (Fig. 1g). Moreover, we confirmed that the ASC speck formation observed was not due to the presence of crystals in the sUA preparations, which can be observed after MSU stimulation in the presence of LPS (Fig. 1h). Indeed, neither phagocytosis inhibition by cytochalasin D (Sup. Fig. 1B) nor blockage of vacuolar H^+^ ATPase system by bafilomicn A (Sup. Fig. 1C) did not lead to decreased IL-1β production by sUA, in opposite to IL-1β production by MSU crystals. Together, these findings identified sUA as a factor able to trigger the NLRP3 inflammasome.

sUA is released following cell death, especially when hypoxic conditions deprive tissues of energy^43^. In an attempt to find the possible source of sUA, we subjected WT macrophages to hypoxic conditions. Hypoxia induced uric acid release (Sup. Fig. 1D) and increased the expression of *IL-1β* (about 6 fold), *Myd88* (about 5 fold) and *Naip1* (about 15 fold), mRNA (Sup. Fig. 1E) by these cells. Hypoxia was reported to reduce mRNA expression of some inflammasome-regulating molecules such as *Nlrp12*^44^ and *Nlrp4*^45^. Accordingly, we found that hypoxia led to IL-1β protein production, a process that can be reversed by uricase treatment (Sup. Fig. 1F). On the other hand, in the absence of either *Nlrp3* or *Myd88*, hypoxic conditions did not lead to IL-1β production (Sup. Fig. 1F).

Differently from other mammals, including mice, humans have lost the capacity to synthesize uricase^15^. For this reason, humans possess tenfold higher concentrations of serum UA than mice (180–400 μΜ versus 18–40 μΜ)^46^,^47^. We then measured IL-1β production by macrophages derived from human monocytes in response to sUA. As shown in supplemental Fig. 1G and 1H, sUA, at a concentration that induces murine IL-1β, in the presence of LPS, there was no production of this cytokine in the supernatant and cell lysate of human macrophages. Indeed, sUA led to reduced LPS-induced TNFα production and increased IL10 production (Sup. Fig. 1H). Despite the consensus that UA released as a result of cell death in both species acts as an inflammatory trigger^3^,^4^, it is possible that pathological concentrations found in mice could not induce an immune response in human cells. These data indicate that 180 μΜ sUA, rather than displaying inflammatory properties, may present an anti-inflammatory activity in human macrophages.

### Soluble Uric Acid Triggers the Inflammasome and Leads to Tissue Damage

**Figure 2:**
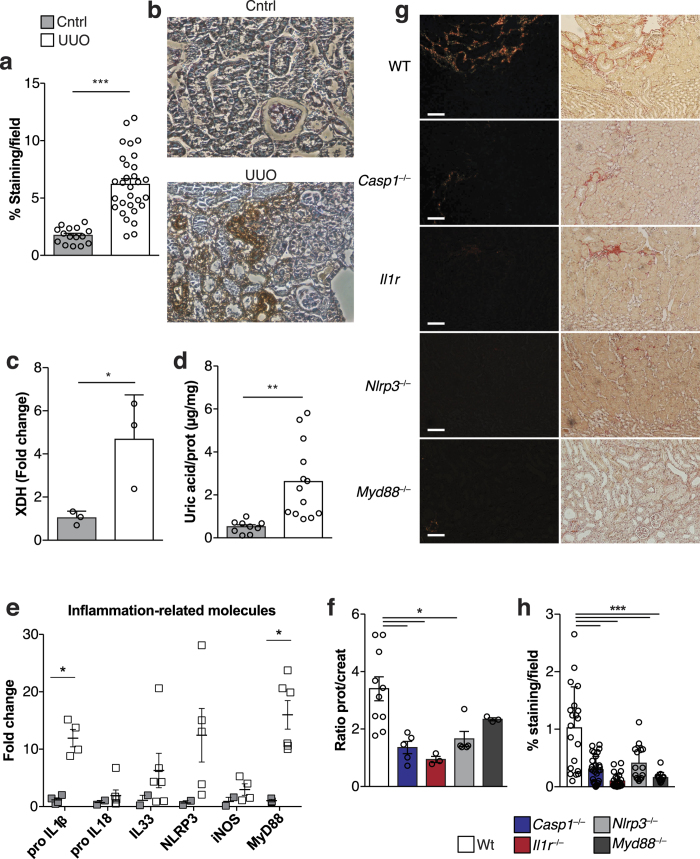
Soluble uric acid triggers the inflammasome and is responsible for renal damage. WT, MyD88^−/−^, NLRP3^−/−^, Casp1^−/−^ and IL1R^−/−^ animals were submitted to ureteral obstruction and renal damage was investigated seven days later. (**a**) Quantitative analysis of the hypoxic area, represented by the proportion of the pimonidazole staining area compared to the total area of the field and (**b)** representative photomicrographs of pimonidazole uptake. (**c**) XDH mRNA, (**d**) sUA and (**e**) inflammasome-related genes in the kidney tissue of WT mice submitted to UUO and sham ones. (**f**) Proteinuria in the urine of obstructed pelvises normalized to that in the urine of the animals before surgery. (**g**) Representative photomicrographs and (**h**) quantification of collagen deposition, as analyzed by Sirius red staining, in the kidneys of WT, Casp1^−/−^, IL1R^−/−^, NLRP3^−/−^ and MyD88^−/−^ mice. In (**a** and **f**), the bars represent 50 μm in all photomicrographs. Sirius red staining and pimonidazole deposition are represented by the percentage of stained area out of the total area in the field. qPCR data were normalized to HPRT expression, and the mean expression in sham mice was considered 1. n = 3 to 13 animals per group in each experiment. *p < 0.05; **p < 0.01; and ***p < 0.001.

In order to investigate the role of sUA *in vivo*, we used an obstructive nephropathy model, unilateral ureter obstruction (UUO), which causes renal fibrosis and inflammation^48^. We observed increased hypoxic areas in obstructed kidneys when compared with sham kidneys (Fig. 2a and b). The energy-deprived state observed in hypoxic conditions can be compensated by the degradation of certain molecules containing high-energy phosphate bonds, leading to increased levels of adenosine, which is subsequently degraded to hypoxanthine, xanthine, and ultimately uric acid^49^. Indeed, we observed an increase in xanthine dehydrogenase (XDH) mRNA expression, the enzyme responsible for transforming purine bases into uric acid^50^ (Fig. 2c). We also observed increased levels of kidneys sUA 7 days post-obstruction (Fig. 2d). The mRNA levels of inflammasome-related cytokines, such as *Il-1β, Il33, Nlrp3* and *Myd88* were also increased in the kidneys 7 days after surgery (Fig. 2e). The hydrostatic pressure ensuing from kidney obstruction can trigger interstitial inflammatory infiltration, indicated mainly by increased numbers of tissue infiltrating-macrophages^48^,^51^. This process contributes toward progressive fibrosis with loss of renal parenchyma and extracellular matrix (ECM) deposition^52^. We therefore observed that deficiency in *Myd88, Nlrp3, Casp1*, or *Il1r* reduced both collagen deposition (Fig. 2f and g) and proteinuria in obstructed pelvises (Fig. 2h) when compared to WT mice.

**Figure 3:**
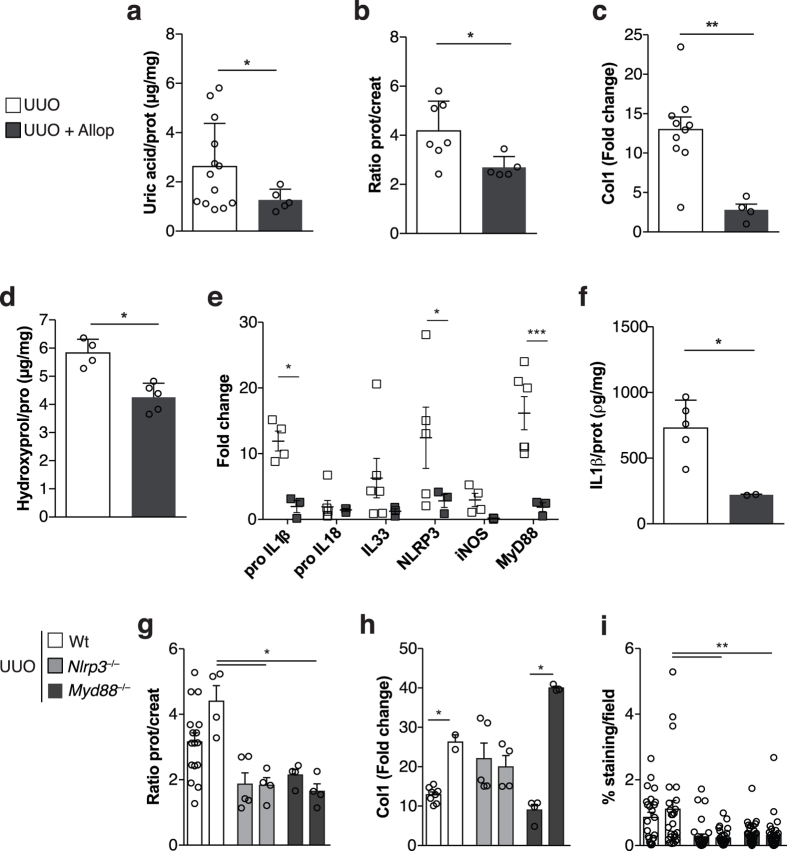
Soluble uric acid is responsible for damage in obstructive nephropathy. Mice were either treated with allopurinol or injected with sUA following ureteral obstruction. (**a–f**) Allopurinol (Alop) treatment in WT mice. (**a**) Soluble tissue uric acid levels normalized by tissue protein. (**b**) Proteinuria in the urine of obstructed pelvises, normalized to that in the urine of the animals before surgery. (**c**) Type 1 collagen mRNA levels and (**d**) hydroxyproline levels normalized by tissue protein. (**e**) Inflammasome-related genes and (**f**) Elisa of IL-1β in obstructed kidneys. (**g–i**) SUA injection into WT, NLRP3^−/−^ and MyD88^−/−^ mice. (**g**) Proteinuria in the urine of obstructed pelvises, normalized to that in the urine of the animals before surgery. (**h**) Type 1 collagen mRNA levels and (**i**) quantification of collagen deposition, as analyzed by Sirius red staining. Sirius red staining deposition is represented by the percentage of stained area out of the total area in the field. qPCR data were normalized to HPRT expression, and the mean expression in sham mice was considered 1. n = 3 to 15 animals per group in each experiment. *p < 0.05.

In an attempt to reverse the fibrotic scenario seen in WT mice submitted to UUO, we administrated allopurinol (Allop), a XDH inhibitor. Allopurinol treatment reduced tissue levels of uric acid (Fig. 3a) and proteinuria in treated UUO mice (Fig. 3b). These mice also displayed decreased type 1 collagen mRNA levels (Fig. 3c) and less hydroxyproline (Fig. 3d), the main amino acid that forms collagen. The mRNA expression of *Il-1β, Il33, Nlrp3, inos* and *Myd88* (Fig. 3e) was also reduced, as well as IL-1β protein (Fig. 3f) in allopurinol treated UUO mice, compared to those not treated. Finally, we injected uric acid into WT, *Myd88*^−/−^ and *Nlrp3*^−/−^ mice submitted to UUO. The injection led to increased protein levels in the urine of obstructed pelvises in WT animals (Fig. 3g). In contrast, proteinuria was not increased in sUA injected-*Myd88*^−/−^ and *Nlrp3*^−/−^. Indeed, uric acid injection increased mRNA expression of type 1 collagen in WT and *Myd88*^−/−^, but not in *Nlrp3*^−/−^ mice (Fig. 3h). In turn, analysis of collagen deposition by sirius red staining demonstrated that uric acid injection led to increased collagen deposition in WT, but not in *Myd88*^−/−^ and *Nlrp3*^−/−^ mice (Fig. 3i). Altogether, our data demonstrate that sUA, released in the context of ureteral obstruction, is responsible for triggering inflammation and for consequent induction of fibrosis, in a *Myd88*- and *Nlrp3*- dependent manner.

### Soluble Uric Acid Triggers the NLRP3 Inflammasome in a Mitochondrial ROS-Dependent Manner

**Figure 4:**
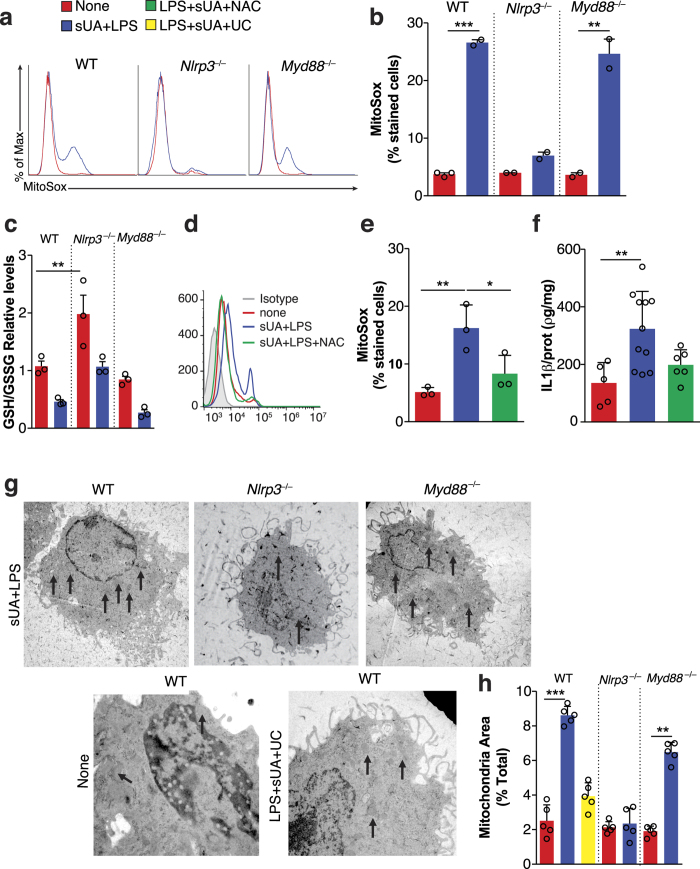
Soluble uric acid triggers the NLRP3 inflammasome and mitochondrial ROS production. WT, NLRP3^−/−^ and MyD88^−/−^ bone marrow-derived macrophages were stimulated with sUA in the presence of LPS (sUA+LPS) and non-stimulated macrophages were used as controls. (**a**) Representative flow cytometry graphs and (**b**) quantification of MitoSox staining in cells under SUA+LPS stimulus or control conditions for 24 h. (**c**) Relative reduced over oxidized glutathione (GSH/GSSG), normalized to total glutathione in WT macrophages under SUA+LPS stimulus or control ones for 24 h. (**d**) Representative flow cytometry graphs and (**e**) quantification of MitoSox staining in WT macrophages in the presence or absence of N-acetyl-l-cysteine (NAC) under SUA+LPS stimulus for 6 h. (**f**) Elisa of IL-1β in supernatants of macrophages analyzed as in (**d**) and (**e**) after 24 h of stimuli. (**g**) Transmission electronic photomicrographs of macrophages under different stimuli. Arrows point to mitochondria. The sUA+LPS representative figures are increased by 5000X and the other two figures are increased by 7000X. (**h**) Quantification of mitochondrial area seen in (**g**). Data are representative of two independent experiments. n = 2 to 11 *p < 0.05; **p < 0.01; and ***p < 0.001. UC = uricase.

In an attempt to address the mechanisms by which sUA promotes NLRP3 inflammasome activation, we analyzed mitochondrial ROS production by staining cells with the mitochondrial-targeting fluorescent probe MitoSOX^53^. Oxidation of MitoSOX Red produces red fluorescence. Contrary to sUA+LPS treated WT and *Myd88*^−/−^, *Nlrp3*^−/−^ macrophages did not display an increase in mitochondrial fluorescence (Fig. 4a and b). We also determined the levels of different forms of glutathione, a ubiquitous thiol-containing tripeptide, implicated in cellular antioxidant defense, which is widely used as a reductant and redox state marker^54^. Relative GSH/GSSG ratios were mostly unchanged in non-stimulated cells except for the *Nlrp3*^−/−^ cells that presented increased ratios (Fig. 4c). After 24 h stimulation with sUA+LPS, WT, *Myd88*^−/−^ and *Nlrp3*^−/−^ macrophages decreased the ratios of GSH to GSSG. In *Nlrp3*^−/−^ cells, however, the ratio of GSH/GSSG decreased to a much lesser extent (Fig. 4c), suggesting a more reduced intracellular environment even after stimulation.

N-acetyl-l-cysteine (NAC), a reductant, inhibits NLRP3 activation in different models^55^,^56^,^57^. Likewise, we observed that NAC decreased MitoSOX fluorescence in WT macrophages after a 6 h stimulation with sUA+LPS (Fig. 4d and e). Also, NAC decreased IL-1β levels (Fig. 4f). Altogether, these data suggest that sUA triggers ROS generation only in the presence of *Nlrp3*, a condition in which the cells present a non-protective redox state. Moreover, treatment with NAC diminished inflammasome activation and consequently, IL-1β production.

In addition to mitochondrial ROS, several inflammasome triggers have been described^58^,^59^. We observed that sUA also activates the NLRP3 inflammasome through ion flux, specifically via potassium efflux, since the inhibition of cellular K^+^ removal in WT macrophages also inhibited IL-1β production (Sup. Fig. 2A). Indeed, sUA+LPS changed the morphology of WT macrophages after 24 h, especially the shape of the plasma membrane (Sup. Fig. 2B). This was not observed in macrophages from *Myd88*^−/−^, *Nlrp3*^−/−^ mice. These data indicate that sUA may trigger the NLRP3 inflammasome in different but complementary manners, such as inducing mitochondrial ROS production, potassium efflux and through changes in cell membrane morphology.

### Soluble Uric Acid Alters Mitochondrial Components

Given the observed changes in mitochondrial redox state, we searched for other functional mitochondrial modifications after sUA+LPS stimulation. sUA+LPS was able to increase mitochondrial area after 24 h in both WT and *Myd88*^−/−^ cells, a process reverted by the addition of uricase (Fig. 4g and h), and suggestive of enhanced mitochondrial biogenesis. There was, however, no increase in mitochondrial area when *Nlrp3*^−/−^ macrophages were exposed to sUA+LPS.

**Figure 5:**
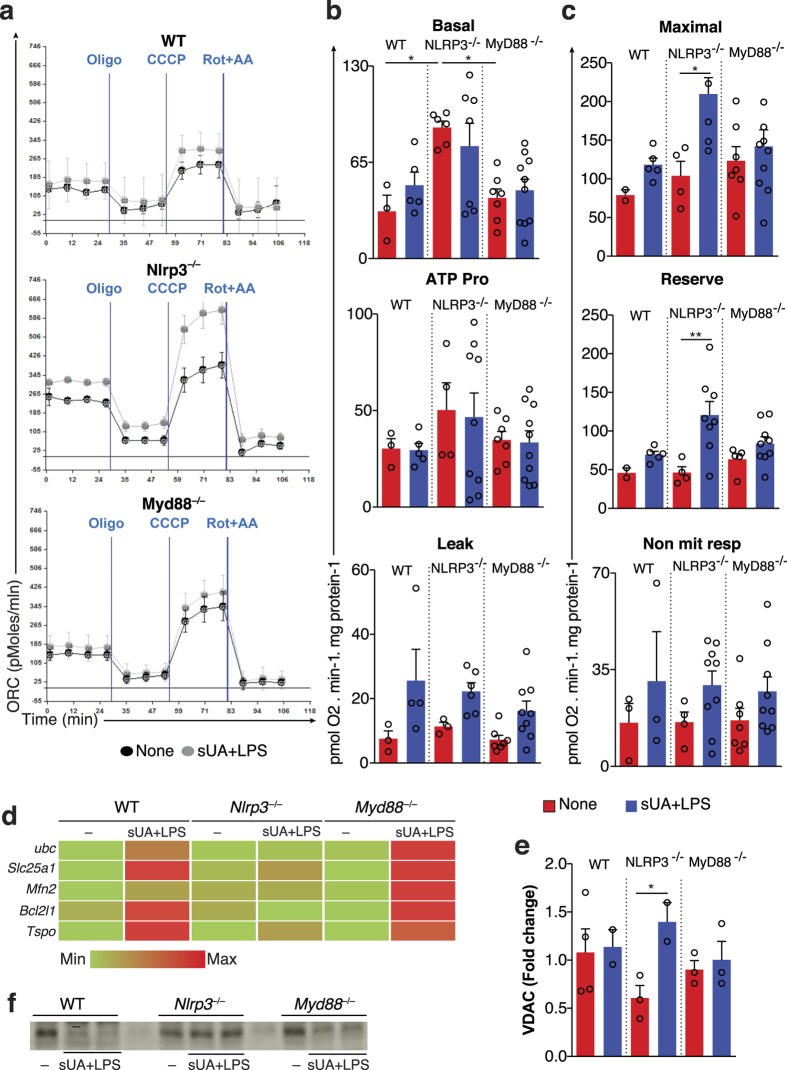
Soluble uric acid promotes mitochondrial modifications in the absence of NLRP3. Bioenergetic profiles of bone marrow-derived macrophages in the presence or absence of sUA+LPS. (**a**) Cells (60,000 per well) were treated with respiratory inhibitors and uncoupler at the following concentrations: oligomycin (1 μgmL), CCCP (5 μΜ) and antimycin A (10 μg/mL) plus rotenone (1 μΜ). (**a**) Representative oxygen consumption rates (OCR) from WT, NLRP3^−/−^ and MyD88^−/−^ macrophages. Gray lines represent sUA+LPS stimulated cells and black lines represent control ones. (**b**, upper graph) Basal OCR (OCR before addition of inhibitors), (**b**, middle graph) ATP production-dependent OCR (basal OCR minus oligomycin-insensitive-OCR), (**b**, lower graph) H^+^ leak (oligomycin-insensitive-OCR), (**c**, upper graph) maximal OCR (highest OCR after CCCP addition), (**c**, middle graph) reserve respiratory capacity (maximal OCR minus basal OCR), and (**c**, lower graph) non-mitochondrial respiration (OCR values in the presence of antimycin A plus rotenone) calculated from experiments such as those depicted in Panel a–c. (**d**) mRNA analysis of mitochondrial membrane component-related genes in different macrophages under sUA+LPS stimulus and non-stimulated conditions. (**e**) VDAC mRNA expression and (**f**) western blotting of VDAC. In (**a–c**), data are representative of three independent experiments and n = 3 to 10. In (**d**), n = 10 in one experiment. In (**e–f**), data are representative of two independent experiments and n = 2 to 4. In (**f**), the blots are cropped in order to improve the clarity and conciseness of the membrane presentation. *p < 0.05; **p < 0.01; and ***p < 0.001.

We attempted next to uncover possible changes in mitochondrial respiration after stimulus with sUA in the presence of LPS by measuring oxygen consumption rates (OCR) of WT, *Myd88*^−/−^, and *Nlrp3*^−/−^ macrophages. Figure 5a show typical OCR traces, and indicate that *Nlrp3*^−/−^ macrophages present increased basal oxygen consumption (the OCR before 30 min) when compared to WT, or *Myd88*^−/−^ cells. sUA+LPS stimulation did not alter basal OCR (Fig. 5b, upper graph). The addition of oligomycin, an inhibitor of mitochondrial ATP synthesis, allowed us to analyze ATP-linked respiration and the proton leak across the inner membrane (Fig. 5b, middle and lower graphs). No differences in ATP-linked oxygen consumption after stimulus with sUA in the presence of LPS were observed, despite the higher ATP-linked OCR in *Nlrp3*^−/−^ macrophages (Fig. 5b, middle graph). On the other hand, sUA+LPS increased the proton leak in all analyzed macrophages (Fig. 5b, lower graph). Additionally, maximal respiratory rates and reserve capacity, measured in the presence of the mitochondrial uncoupler CCCP, were larger after sUA+LPS stimulus in *Nlrp3* deficiency, but not in *Myd88*^−/−^ and WT macrophages (Fig. 5c, upper and middle graphs). The non-mitochondrial OCR was uncovered using rotenone to inhibit Complex I and antimycin A to inhibit Complex III^60^. We observed that sUA+LPS increased non-mitochondrial respiration in all analyzed macrophages (Fig. 5c, lower graph). Overall, our data demonstrate that among all macrophages analyzed, only *Nlrp3*^−/−^ cells increased maximal and reserve OCR under sUA+LPS stimulus. We next increased the maximal OCR of WT macrophages by using CCCP at low doses (Sup. Fig. S3A–C). We observed no differences in IL-1β production from WT macrophages stimulated with sUA+LPS treated or not with of CCCP (Sup. Fig. S3D and E). Our data suggests the responsiveness to sUA is not directly dependent on mitochondrial oxygen consumption rates.

Due to the observed changes in mitochondrial function, we searched for differential expression of mitochondrial membrane component-related genes (Fig. 5d). Since redox changes can alter protein turnover^61^,^62^, we focused on ubiquitin c (Ubc) and observed an upregulation of Ubc mRNA levels only in WT and *Myd88*^−/−^ macrophages after stimulation with sUA in the presence of LPS. Indeed, WT and *Myd88*^−/−^, but not *Nlrp3*^−/−^ macrophages, presented increased tricarboxylate transport protein (Slc25a1) mRNA, a citrate carrier protein necessary for the generation of mitochondrial oxidative stress upon uric acid stimulation^63^,^64^ (Fig. 5d). We also investigated the mRNA expression of some outer mitochondrial membrane proteins after sUA+LPS stimulus. Mitofusin-2 (Mfn2)^65^,^66^,^67^ and Bcl-2-like protein 1 (Bcl2l1)^68^ mRNA levels did not increase in *Nlrp3*^−/−^ cells. Indeed, the translocator protein (Tspo), a protein that binds to the voltage-dependent anion channel (VDAC) and interferes with redox balance^69^,^70^,^71^ was upregulated after sUA+LPS stimulus in all macrophages analyzed, but in a more pronounced way in WT and *Myd88*^−/−^ cells. Ultimately, VDAC, a protein involved in the transport of ATP, calcium and other metabolites across the mitochondrial outer membrane^72^,^73^, and related to integrity of this organelle in the context of NLRP3 activation^74^, was upregulated in *Nlrp3*^−/−^ macrophages upon sUA+LPS (Fig. 5e). Consistently, protein expression analyses demonstrated that sUA+LPS stimulus led to reduced VDAC expression in WT and *Myd88*^−/−^ macrophages, while *Nlrp3*^−/−^ macrophages displayed similar levels of VDAC protein to non-stimulated cells (Fig. 5f). A set of differentially expressed mitochondrial components is shown (Sup. Fig. S4). It is predicted that *Nlrp3* deficiency promotes changes in mitochondrial dynamics, a feature that has been proposed to be important under stressful conditions, especially under inflammasome activation.

## Discussion

The study of uric acid reveals an intriguing aspect of evolution. Great Apes lost the uricase gene, a loss that may be related to a survival advantage^16^, giving rise to high-energy storage ability^75^. It is also accepted that, in some contexts, uric acid is a major antioxidant that protects cardiac, vascular, and neural cells from oxidative injury^76^,^77^. However, hyperuricemia, even without crystal deposition^13^, is strongly associated with disorders such as kidney disease, and hypertension^78^. A common pathogenic feature for all of these disorders is, paradoxically, the involvement of redox imbalance^79^,^80^,^81^. We evaluated the role of soluble uric acid and found that it displays an inflammatory role in murine cells. We, therefore, do not discard the hypothesis that the observed effects could be caused by microcrystals of uric acid, non-visualized by laser scanning confocal microscopy combined with reflection microscopy^7^ or polarizing microscopy^82^. Accordingly, the role of sUA was confirmed using an *in vitro* assay in which we demonstrated that sUA stimulated the dose- and time-dependent production of inflammasome-related molecules in the presence of *Nlrp3* and *Myd88*. We confirmed our results using a model of renal disease in which we observed increased levels of sUA in the absence of crystals.

Elevated serum uric acid is associated with an increased prevalence of chronic kidney disease (CKD)^83^. The end stage of CKD, renal fibrosis, is the result of an injury that leads to the production of ECM components. Once this production is continuous, ECM deposition becomes massive and, associated with the uncontrolled apoptosis of tubular cells, results in tubular atrophy^84^. Matrix remodeling and cellular stress result in the release of molecules that ultimately activate an inflammatory response through innate-sensing receptors^85^,^86^,^87^. Until now, it was unclear how these innate immune receptors relate to fibrosis. We hypothesized that this connection involves inflammasome signaling. We observed that renal damage seen in UUO is dependent on NLRP3, MyD88, caspase-1 and IL-1 receptor. Additionally, we confirmed that uric acid is released after hypoxic conditions^88^, observed, for instance, after UUO, a model of urinary flow obstruction^89^,^90^. Indeed, increased sUA release correlated with increased kidney damage at seven days post UUO. Ultimately, inflammasome activation and IL-1β production lead to extracellular matrix deposition and kidney fibrosis^91^,^92^.

Two signals are required for inflammasome activation: the first signal, mediated by TLR, must be capable of inducing increased expression of pro-IL-1β; the second signal would be responsible for cleavage of pro-IL-1β in its active form^21^. There are several hypotheses for the second signal activation and further investigations are necessary in order to find whether is there any common component for all second signals described, for example, the release of ROS^93^. We propose that the mechanism underlying sUA-induced inflammation is dependent on redox state changes and mitochondrial ROS production, in accordance with data demonstrating that high concentrations on uric acid may induce oxidative stress^94^. We observed that *Nlrp3*^−/−^ macrophages present an intrinsically protective redox state and this could be the reason why macrophages did not produce ROS after sUA+LPS stimulus. *Nlrp3*^−/−^ macrophages also presented higher maximal and reserve oxygen consumption rates when compared to *Myd88*^−/−^ and WT cells, which can be explained by the fact that ROS, chronically, lead to reduction in maximal and reserve capacity^95^. The increase in maximal oxygen consumption rates after CCCP stimulation for 24 hours at low doses, however, was not sufficient to prevent IL-1β production in WT macrophages. It seems that the decreased inflammasome activation is related to mitochondrial changes, but not to the increase in maximal oxygen consumption rates. Among the changes that mitochondria can undergo, organelle dysfunction has been demonstrated to be an NLRP3 inflammasome activator^96^. Indeed, mitochondria have multiple mechanisms that allow them to activate signaling pathways in the cytosol including altering in AMP/ATP ratios, the release of TCA cycle metabolites, as well as the localization of immune regulatory proteins to the outer mitochondrial membrane^42^.

*Nlrp3*^−/−^ macrophages did not show changes in the expression of some mitochondrial membrane compound-related genes. On the other hand, VDAC mRNA increased after sUA+LPS stimulus in *Nlrp3*^−/−^, but not in WT or *Myd88*^−/−^ macrophages. VDAC, a protein related to the NLRP3 inflammasome^74^, is known to regulate mitochondrial ROS production^97^. The large number of cysteines in VDAC is believed to confer protection from ROS build-up in mitochondria^72^. It could be possible that *Nlrp3* deficiency prevent cell response to sUA by removing the ability to produce ROS under stimulation. However, new studies are needed to better evaluate the flux of ions and ROS through mitochondrial membranes and the potential of mitochondrial membrane in the context of *Nlrp3* deficiency, under different inflammasome stimuli.

Altogether, our results provide evidence that uric acid, in its soluble form, is responsible for increasing IL-1β production in an *Nlrp3*- and *Myd88*-dependent manner. The NLRP3 inflammasome stimulation by sUA is accompanied by cellular redox state changes, increasing in mitochondria area and mitochondrial ROS production. The observation that inflammasome activation can be triggered by sUA may have profound implications for fibrotic-related diseases. We propose that future therapeutic strategies for renal fibrosis, for instance, should be based on blocking sUA or inhibiting its recognition by phagocytes.

## Material and Methods

### Animal studies

*Lyz*^*Cre*^*Nlrp3*^*flox*^ or *Itgax*^*Cre*^*Nlrp3*^*flox*^ animals were generated by crossing *Lyz*^*Cre*^ or *Itgax*^*Cre*^ mice with *Nlrp3*^*flox/flox*^ mice. Male *Myd88*^−/−^, *Nlrp3*^−/−^, *Casp1*^−/−^, *Il1r*^−/−^, *Lyz*^*Cre*^*Nlrp3*^*flox*^ or *Itgax*^*Cre*^*Nlrp3*^*flox*^ animals and control C57Bl/6 mice, aged 6 to 8 weeks, were bred and housed in a pathogen-free facility at the University of São Paulo. On day 0, unilateral ureteral obstruction (UUO) was performed by complete ligation of the right ureter. Seven days later, mice were sacrificed for biochemical, histological, protein and genetic analyses. Allopurinol (Schein Pharmaceutical, Florham Park, NJ, USA) was administered in the drinking water at 13 mg/dL to one cohort from day 0 until day 7. Uric acid (120 mg/kg *i.p.*) was injected intraperitoneally on day 0. All procedures were approved by the local ethics committees at the University of São Paulo (Document 45/2009). All experiments were performed in accordance with relevant guidelines and regulations.

### Soluble uric acid preparation

In experiments requiring soluble uric acid, media was pre-warmed (37 °C), and uric acid (Ultrapure, Sigma; 180 μΜ) was added. The mixture was warmed again (37 °C, 30 minutes) and sterilized through 0.20 μm filters. Crystals were not detectable under these conditions (polarizing microscopy), nor did they develop during cell incubation.

### Murine cells

Bone-marrow derived macrophages (BMDMs) were obtained by culturing bone marrow cells from 6- to 8-week old mice in DMEM supplemented with 20% L929-derived medium and 10% FBS. Seven days later, BMDMs were collected and plated. The inflammasome reporter macrophage stably transduced with constructs for the expression of mCerulean-tagged ASC has been described previously^98^.

### Differentiation of human monocyte-derived macrophages *in vitro*

Human peripheral blood mononuclear cells (PBMCs) were obtained by a centrifugation gradient in Ficoll-Paque (Dominique Dutscher). Subsequently, PBMCs were centrifuged in 51% PBS-diluted Percoll (GE Healthcare Life Sciences) and monocytes (>70% purity) were adhered in six well plates during 2 hours. After removal of non-adherent cells by washing the plates, monocytes were differentiated into macrophages in RPMI medium (Gibco, Grand Island, NY, USA) supplemented with 10% FCS (Gibco) plus antibiotics and antimycotics (100 U/ml penicillin, 100 g/ml streptomycin, and 25 g/ml amphotericin; Gibco) in the presence of GM-CSF (50 ng/ml; R&D Systems, Minneapolis, MN, USA) for seven days.

### Renal function outcomes

Urinary protein/creatinine ratios were measured in samples collected from the pelvis of obstructed mice 7 days post-surgery. All samples were analyzed through colorimetric assays using commercially available kits for creatinine and protein measurement (Labtest, Minas Gerais, Brazil). Normalization was achieved by dividing the protein/creatinine ratio at day 7 by the ratio before surgery in each group of animals. Uric acid was measured in the kidney 7 days post-surgery (Labtest, Minas Gerais, Brazil). The results are presented as μg uric acid/mg of total protein, which was measured using the Bradford assay (Bio-Rad, Hercules, CA).

### Sirius red staining

Kidneys were harvested and placed in 10% buffered formaldehyde for fixation. In short, the slides were deparaffinized, rehydrated, and immersed in saturated picric acid solution for 15 minutes and then in Picrosirius for 20 minutes. Counterstaining was performed using Harris hematoxylin. Picrosirius-stained sections were analyzed using an Olympus BX50 microscope and camera. Manual photographs were taken of the renal cortex magnified at 40X and observed under polarized light. Images of at least 10 different fields in each slide were taken, and structures such as the glomeruli, sub capsular cortex, large vessels, and medulla were excluded. For the morphometric analysis, Java Image J software image processing and analysis were used. The results were represented as percentages and refer to the proportion of stained volume to total cortical interstitial volume.

### Tissue hypoxia staining

To probe for kidney hypoxia, 60 mg/kg of pimonidazole (Chemicon, Temecula, CA) were injected intraperitoneally^99^. Staining was performed on paraffin sections using a microwave-based antigen retrieval technique. Sections were then incubated with the labeled polymer (Dual Link System-HRP, Glostrup, Denmark, DAKO). Staining was completed by incubating sections for 1 to 3 minutes with 3,3-0-diaminobenzidine substrate-chromogen, which results in a brown-colored precipitate at the antigen site. Then hematoxylin counterstaining was performed. Images of at least 10 different fields in each slide were taken and the result of the analysis was represented as percentages and refers to the proportion of stained volume to total volume.

### Transmission electron microscopy

Macrophages were fixed in PBS containing 4% PFA, 2% glutaraldehyde and 0.1 M cacodylate, pH 7.4 overnight at 4 °C. Samples were rinsed twice in PBS, and were postfixed in 1% osmium tetroxide (OsO4) for 2 hours. After that, cell pellets were placed in 0.5% uranile acetate in distilled water with sucrose 1% at 4 °C for 24 h. Cells were dehydrated in a series of alcoholic solutions from 70 to 100%, followed by two immersions in propylene oxide for 20 min each, propylene oxide and araldite infiltration for 12 h, at room temperature on a shaking platform. Subsequently, cells were incubated overnight in Spurr™ resin 1:1 mixed with 100% ethanol, before embedding in Spurr™ resin. Semithin sections were stained with toluidine blue and examined in the light microscope. Ultrathin sections were double stained with 2% uranyl acetate and lead citrate. Observation was performed using a JEOL-JEM-100 CXII Transmission Electron Microscopy (TEM).

### Quantitative real-time PCR

RT-PCR was performed using the Taqman real-time PCR assay (Applied Biosystem, USA) for the following molecules: *Hprt* (Mm00446968_m1), *Il-1β* (Mm00434228_m1), *Myd88* (Mm00440338_m1), *Xdh* (Mm00442110_m1), *Il18* (Mm00434226_m1), *Il-33* (Mm00505403_m1), *Type 1 collagen* (Mm00801666_g1), *inos* (Mm00440502_m1) and *Nlrp3* (Mm00840904_m1). Cycling conditions were as follows: 10 minutes at 95 °C, followed by 45 20 second cycles at 95 °C, 20 seconds at 58 °C, and 20 seconds at 72 °C. Analysis was performed using Sequence Detection Software 1.9 (SDS). mRNA expression was normalized to HPRT expression.

### Hydroxyproline quantification

Hydroxyproline quantification was determined according to Reddy *et al*.^100^. Briefly, standard hydroxyproline (St. Louis, USA, Sigma) aliquots or kidney samples were hydrolyzed in sodium hydroxide (2 N final concentration). The hydrolyzed samples were then mixed with a buffered chloramine-T reagent, and oxidation was allowed to proceed for 25 minutes at room temperature. The chromophore was then developed with the addition of Ehrlich’s reagent, and the absorbance of the reddish-purple complex was measured at 550 nm using a spectrophotometer. Absorbance values were plotted against the concentration of standard hydroxyproline, and the presence of hydroxyproline was determined using the standard curve.

### Cytokine profile

Total renal IL-1β protein from the culture supernatant were measured using IL-1β (R&D Systems, Minneapolis, MN, USA) Emax immunoassay systems (Promega, Madison, USA), according to the manufacturer’s instructions.

### Confocal imaging

Confocal Laser Scanning Microscopy (CLSM) was performed with a Leica TCS SP5 SMD confocal system (Leica Microsystems). For live imaging, the temperature was maintained at 37 °C with 5% CO_2_ using an environmental control chamber (Life Imaging Services and Solent Scientific). Images were acquired using a 63X objective at time points noted in the figures or figure legends and analyzed using the LAS AF version 2.2.1 (Leica Microsystems) or Volocity 6.01 software.

### Glutathione levels

The concentrations of GSH and GSSG, as well as total glutathione, were determined using a colorimetric DTNB assay, as described in ref. 101. GSH reacts with DTNB producing TNB and GS-TNB, which is reduced back to GSH by GR. This process releases TNB, detected by absorbance at 412 nm.

### Oxygen consumption rates

An hour before oxygen consumption measurements, cell media was replaced by assay media (2 mM glucose, 0.8 mM Mg^2+^, 1.8 mM Ca^2+^, 143 mM NaCl, 5.4 mM KCl, 0.91 mM NaH_2_PO_4_, and 15 mg/mL Phenol red) for 60 min at 37 °C (no CO_2_) before loading into the Seahorse Bioscience XF96 extracellular analyzer^102^. During these 60 min, the ports of the cartridge containing the oxygen probes were loaded with the compounds to be injected during the assay (75 μL/port) and the cartridge was calibrated. Basal respiration was recorded for 30 min, at 5 min intervals, until system stabilization. CCCP was used at final concentrations of 5 mM and injected with sodium pyruvate (Sigma) at a final concentration of 5 mM. Oligomycin and antimycin A were used at final concentrations of 1 and 10 μg/mL, respectively. Rotenone was used at a concentration of 1 μM. All respiratory modulators were used at ideal concentrations titrated during preliminary experiments (not shown). A typical OCR chart is displayed, where OCR represents the percentage of basal respiration.

### Statistics

The data were described in terms of the mean and S.E.M. when three or more experiments were performed and S.D. when two different experiments were performed. Differences between groups were compared using ANOVA (with Tukey’s post-test) and Student’s *t*-test. Significant differences were regarded as p < 0.05. All statistical analyses were performed using GraphPad PRISM 6.01 (La Jolla, CA, USA).

## Additional Information

**How to cite this article**: Braga, T. T. *et al*. Soluble Uric Acid Activates the NLRP3 Inflammasome. *Sci. Rep.* **7**, 39884; doi: 10.1038/srep39884 (2017).

**Publisher's note:** Springer Nature remains neutral with regard to jurisdictional claims in published maps and institutional affiliations.

## Supplementary Material

Supplemental Information
