# Supplementary material for: Soluble Uric Acid Activates the NLRP3 Inflammasome
Source: Sci Rep. 2017 Jan 13;7:39884. doi: 10.1038/srep39884 (PMC5233987; doi:10.1038/srep39884)
Supplement: Supplemental Information [file srep39884-s1.pdf]

Soluble Uric Acid Activates the NLRP3 Inflammasome

Tarcio Teodoro Braga, Maria Fernanda Forni, Matheus Correa-Costa, Rodrigo Nalio Ramos, Jose Alexandre Barbuto, Paola Branco, Angela Castoldi, Meire Ioshie Hiyane, Mariana Rodrigues Davanso, Eicke Latz, Bernardo S Franklin, Alicia J. Kowaltowski, Niels Olsen Saraiva Camara.

Running title:

Uric acid, renal fibrosis, ROS and NLRP3

*Corresponding author: Tarcio Teodoro Braga. PhD.

Laboratory of Transplantation Immunobiology, Department of Immunology, Institute of Biomedical Sciences IV, University of São Paulo (USP), São Paulo, Brazil. Email: tarcio.tb@gmail.com. Phone: (+5511) 30917388, Fax: (+5511)30917224.

Supporting Figure S1

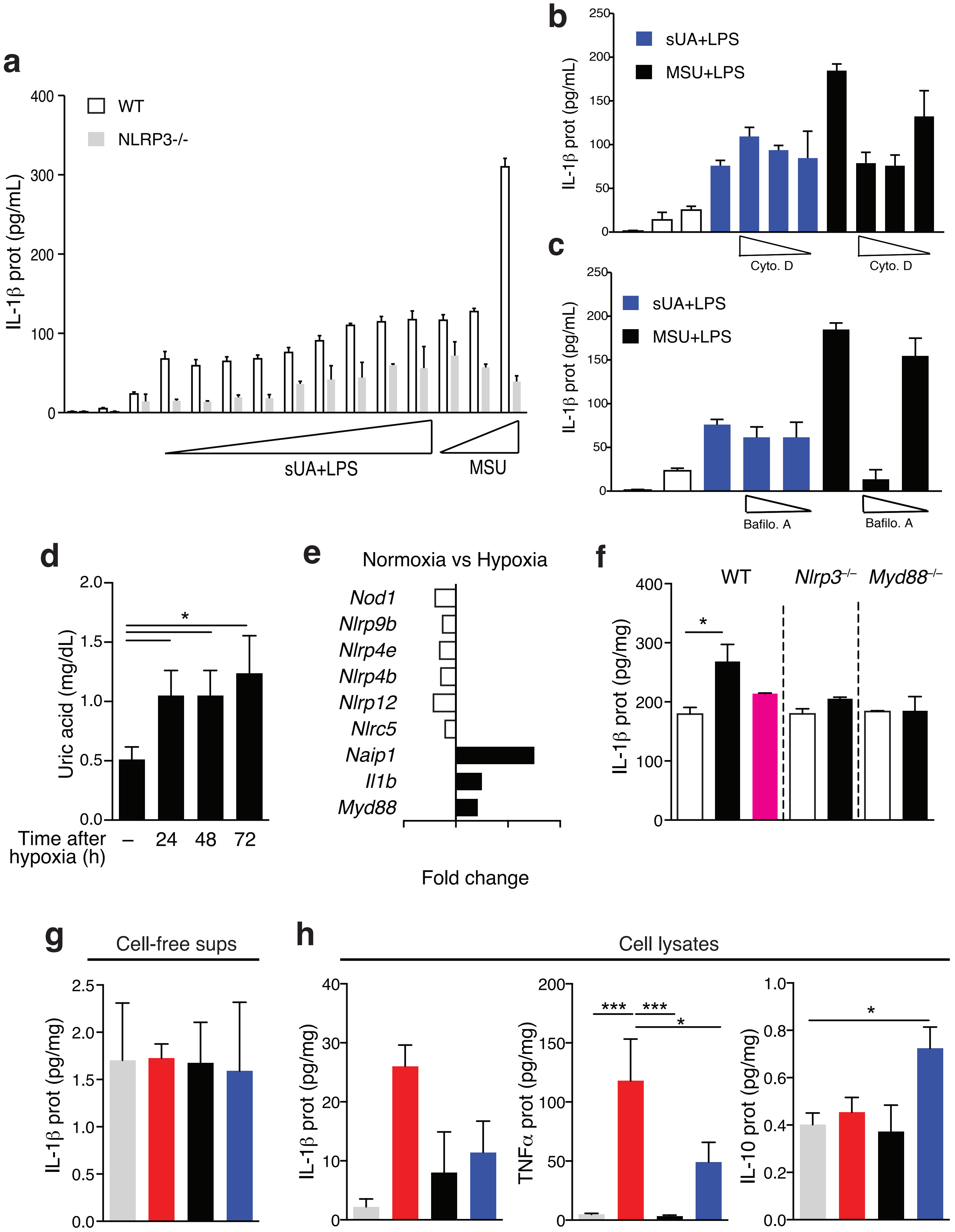

Supporting Figure S2

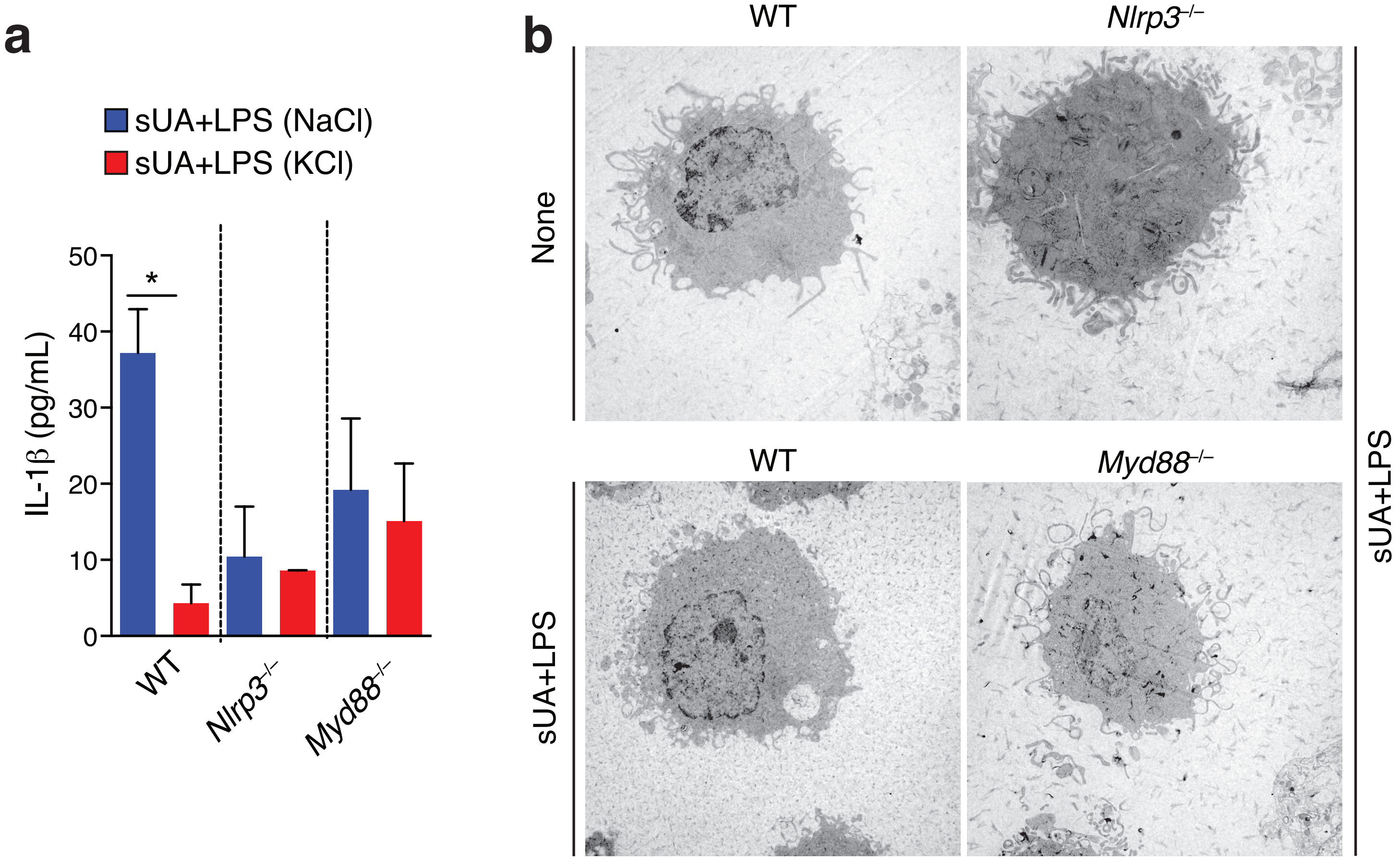

Supporting Figure S3

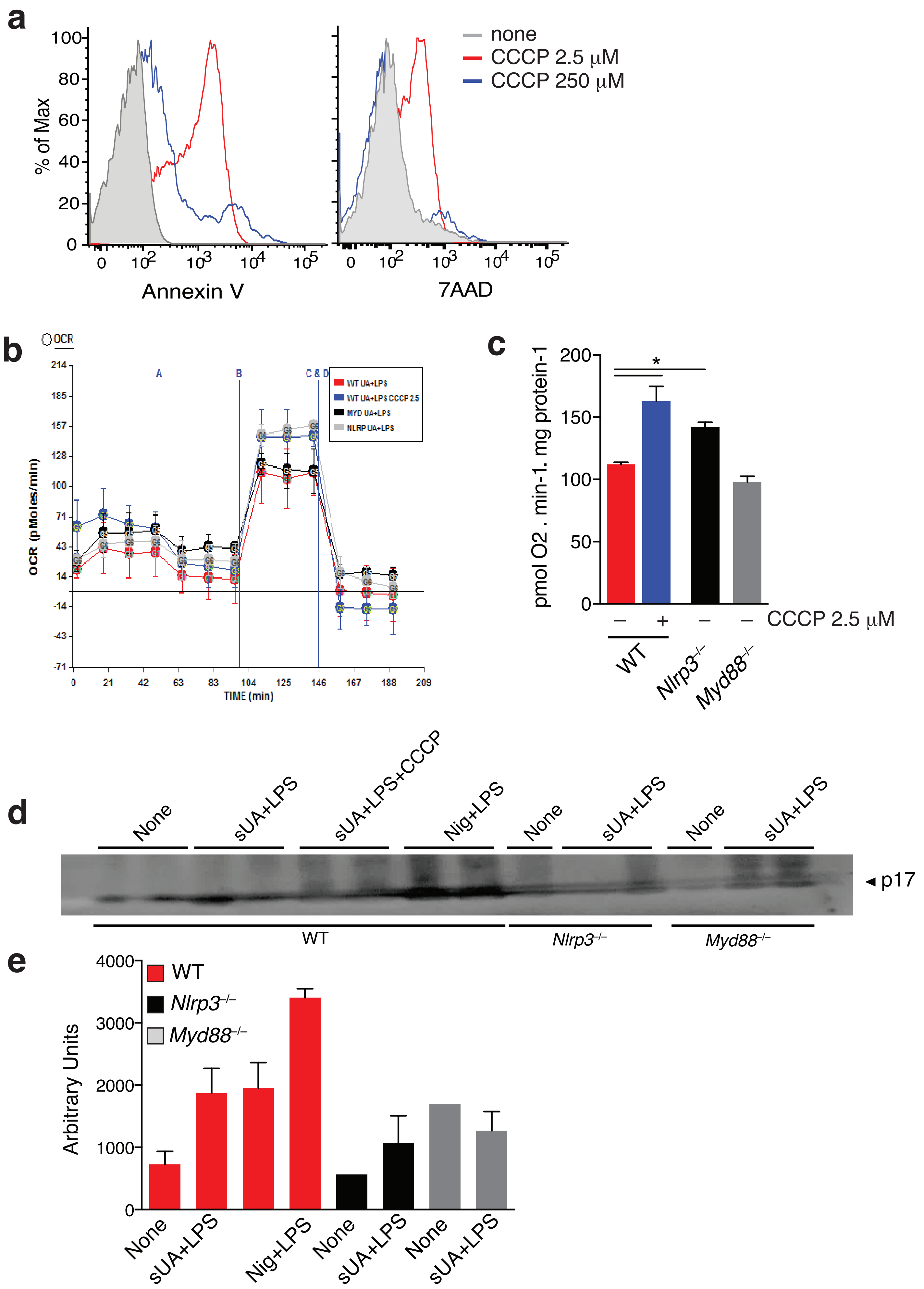

Supporting Figure S4

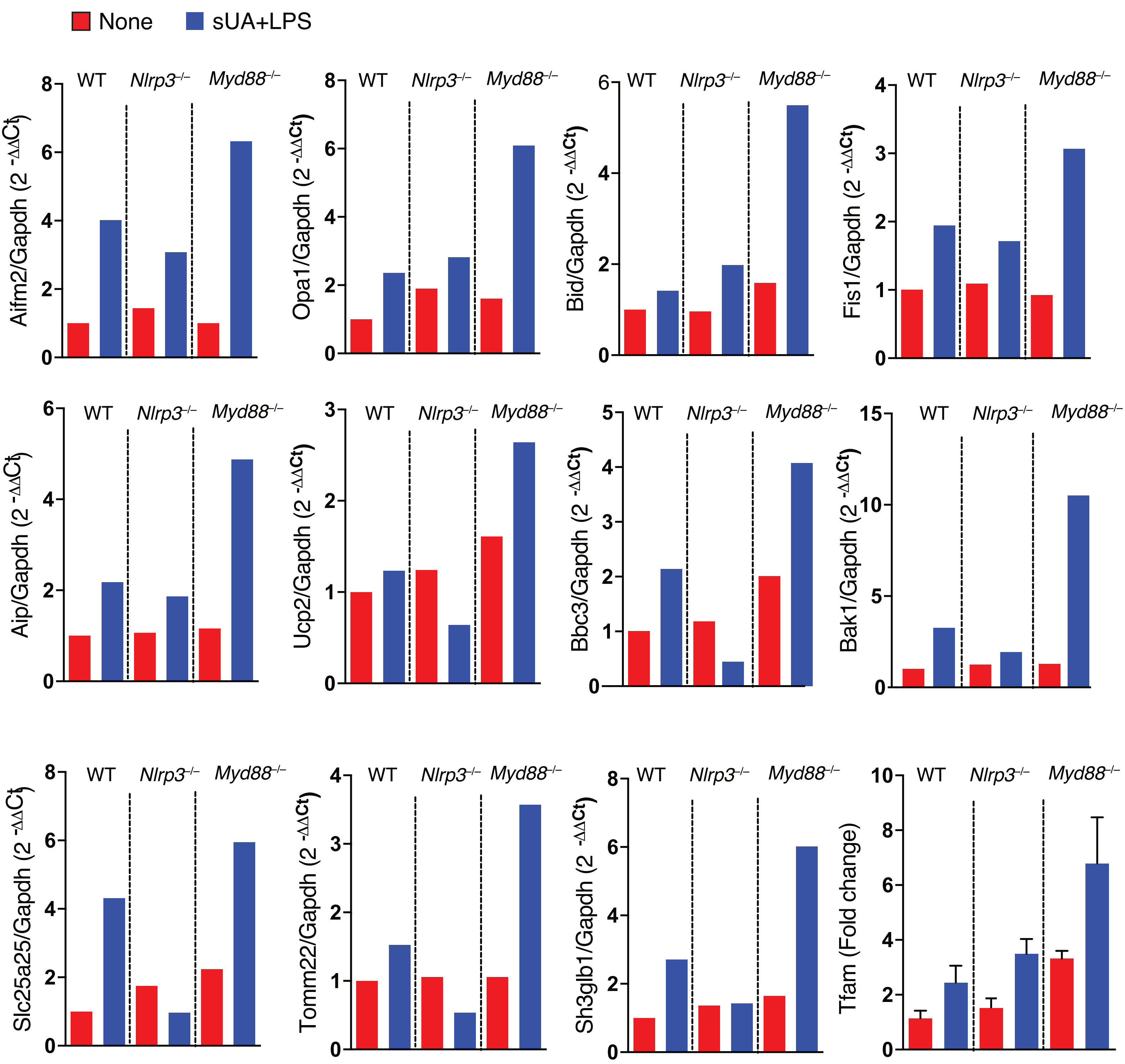

SUPPLEMENTAL FIGURE LEGEND

Supplemental Figure 1: Soluble uric acid activates inflammasome in a phagocytosis-independent way, is derived from hypoxic conditions and does not activate human macrophages. (A) Dose-response effect of soluble uric acid and MSU crystals, in the presence of LPS, on IL-1β production into WT and NLRP3 deficient macrophages stimulated for 24 hours. The concentration range of soluble uric acid goes from 36 to 900 μM, while the concentration of MSU crystal goes from 250 to 1000 μg/mL. (B) Effect of cytochalasin D on IL-1β production into WT macrophages stimulated for 6 hours upon stimulus with sUA+LPS or MSU+LPS. The concentrations of cytochalasin D are 2.5 μM, 1.3 μM or 0.6 μM. (C) Effect of bafilomycin A on IL-1β production into WT macrophages stimulated for 6 hours upon stimulus with sUA+LPS or MSU+LPS. The concentrations of bafilomycin A are 250 nM or 50 nM. (D) SUA levels in the supernatant of macrophages after 24, 48 and 72 hours of hypoxia and in the supernatant of cells under control normoxia. (E) Genes differentially expressed in macrophages after 24 h of hypoxia. Black bars represent upregulated genes and white bars represent downregulated genes in cells after hypoxia when compared to cells in normoxic conditions. (F) IL-1β quantification in the supernatant of macrophages analyzed in B. (G) IL-1β quantification in the supernatant of human monocyte-derived macrophages under stimuli indicated in the bottom of the graph. (H) IL-1β, TNFα and IL10 quantification in the cell lysate of human monocyte-derived macrophages under stimuli indicated in the bottom of the graph. Data are representative of three independent experiments and n=5. *$p < 0.05$; and ***$p < 0.001$.

Supplemental Figure 2: Soluble uric acid activates inflammasome through overlapping mechanisms. (A) IL-1β quantification in the supernatant of WT, MyD88-/- and NLRP3-/- macrophages after SUA+LPS stimulation in the presence or absence of KCl (70mM). (B) Transmission electronic photomicrographs of macrophages under different stimuli. The figures are increased by 5000X. Data are representative of two independent experiments. n=5. *$p < 0.05$.

Supplemental Figure 3: The increase maximal OCR is not able to inhibits inflammasome activation by soluble uric acid. (A) WT macrophages were stained with Annexin V and 7AAD after 24 hours of 2.5 μM and 250 μM CCCP stimulation or the control non-stimulated cells. (B) WT, MyD88-/- and NLRP3-/- macrophages (60,000 per well) after SUA+LPS stimulation and WT macrophages stimulated with SUA+LPS in the presence of 2,5 μM CCCP for 24 hours were treated with respiratory inhibitors and uncoupler at the following concentrations: oligomycin (1 μg/mL), CCCP (5 μM) and antimycin A (10 μg/mL) plus rotenone (1 μM). Representative oxygen consumption rates (OCR) are shown. (C) Maximal OCR (highest OCR after CCCP addition). (D) Western blotting in supernatants of WT, MyD88-/- and NLRP3-/- macrophages after SUA+LPS stimulation and WT macrophages stimulated with SUA+LPS in the presence of 2,5 μM CCCP for 24 hours. (E) Quantification of IL-1β western blotting. Data are representative of two independent experiments. n = 5. *$p < 0.05$.

Supplemental Figure 4: Soluble uric acid alters mitochondrial membrane component-related genes. (A) Aifm2, (B) opa1, (C) Bid, (D) Fls1, (E) Alp, (F) Ucp2, (G) Bbc3, (H) Bak1, (I) Slc25a25, (J) Tomm22, (K) Sh3glb1 and (L) Tfam mRNA quantification in WT, MyD88-/- and NLRP3-/- macrophages under SUA+LPS stimulus and non-stimulated ones. In A-K, n = 10 in one experiment and data were normalized to GAPDH expression, and the mean of the control condition was considered 1. In L, data is representative of two independent experiments and n = 5. In L, qPCR data was normalized to HPRT expression, and the mean of the control condition was considered 1.
